# A ventral hippocampus to nucleus accumbens pathway regulates impulsivity in male but not female rats

**DOI:** 10.1038/s41386-026-02477-0

**Published:** 2026-06-26

**Authors:** Molly E. Klug, Léa Décarie-Spain, Logan Tierno Lauer, Bo W. Sortman, Bingxin Mo, Alicia E. Kao, Olivia P. Moody, Nicolas R. Morano, Haoyang Huang, Don B. Arnold, Emily E. Noble, Alexander W. Johnson, Scott E. Kanoski

**Affiliations:** 1 Human and Evolutionary Biology Section, Department of Biological Sciences, Dornsife College of Letters, Arts and Sciences, University of Southern California, Los Angeles, CA USA; 2 Department of Psychology, Michigan State University, East Lansing, MI USA; 3 Molecular and Computational Biology Section, Department of Biological Sciences, Dornsife College of Letters, Arts and Sciences, University of Southern California, Los Angeles, CA USA; 4 Nutritional Sciences, College of Family and Consumer Sciences, University of Georgia, Athens, GA USA

**Keywords:** Obesity, Motivation, Reward

## Abstract

Heightened impulsivity is associated with substance abuse disorders, gambling, and obesity. The ventral hippocampus (vHPC) has recently been linked with impulse control, yet the neurobiological and behavioral mechanisms through which this occurs are unknown. A subset of vHPC neurons projects to the nucleus accumbens shell (ACBsh), a brain region known for regulating reward and motivation. Here, we evaluated the role of vHPC-ACBsh signaling in food-directed impulsivity using fiber photometry and transsynaptic chemogenetic and behavioral approaches. Male and female rats were trained in the differential reinforcement of low rates of responding (DRL) test of impulsive action, where they learn to withhold lever presses for 20 s to obtain a palatable food reward. Photometry recordings during DRL revealed elevations in calcium-dependent activity in the vHPC during the 5 s immediately prior to a non-impulsive vs. an impulsive response in males but not females. Chemogenetic silencing of ACBsh-projecting vHPC neurons elevated impulsive responses in DRL relative to vehicle treatment in males but not females, yet had no effect on home cage food intake, operant-based motivation to work for palatable food, impulsive choice, or anxiety-like behavior. To determine whether this impulse control circuit requires vHPC->ACBsh communication independent of collateral targets of ACBsh-projecting vHPC neurons, we utilized a novel transsynaptic viral approach to selectively silence ACB neurons that receive synaptic glutamatergic signaling from the vHPC. Results reveal that inhibition of ACBsh neurons receiving vHPC signaling elevates impulsive action in the DRL task relative to vehicle treatment. Collective results reveal a sex-specific hippocampal-striatal circuit that regulates impulsivity.

## Introduction

Impulsivity, characterized by acting with little to no forethought for action consequences, has been implicated in obesity, binge-eating disorder (BED), compulsive gambling, and various substance abuse disorders [1–9]. One hypothesis for the etiology of the obesity crisis, which affects over 40% of adults in the US [10], centers around deficits in behavioral inhibition and increased reward-salience, which in an obesogenic environment can lead to impulsive food-directed behaviors resulting in excessive caloric consumption [5, 11, 12]. Consistent with this hypothesis, obesity is associated with lower inhibitory control scores, an outcome predictive of success in behavioral weight loss intervention programs [1]. Further, deficits in behavioral inhibition and impulse control are strongly linked with BED, as well as both childhood and adult obesity [1, 2, 13, 14].

Impulsivity, while a complex and multifaceted personality trait, can be divided into two distinct subtypes: behaviors resulting from a failure to suppress an inappropriate action (impulsive action) and behaviors wherein the choice between two or more options is made without proper consideration of future consequences (impulsive choice). While the neural circuitry regulating impulse control is poorly understood, emerging evidence indicates that impulsive actions and impulsive choices are modulated by both overlapping and distinct neural substrates [15, 16]. Furthermore, there is significant support for sexual dimorphism in trait impulsivity, with males being generally more susceptible than females [17–20]. Thus, understanding the neural substrates underlying impulsivity must involve both behavioral task- and sex-specific evaluations.

The hippocampus (HPC), a brain region crucial for the encoding, consolidation, and retrieval of episodic and spatial memories, has recently been linked to regulating food-directed impulse control [21, 22], as well as the control of eating behavior more generally. HPC damage is associated with impaired satiety [23, 24]. and selective HPC lesions in rodents leads to increased food intake and body weight gain [25]. Further, acute inactivation of either the dorsal or ventral HPC subregion immediately after a meal increases subsequent food consumption in rodents [26, 27], and an orexigenic hippocampal subnetwork associated with dysregulated eating and body mass index in humans was recently identified [28]. However, the neural systems through which the hippocampus regulates impulsive responding for palatable foods remain poorly understood.

A putative downstream site-of-action for HPC-mediated impulse control is the nucleus accumbens (ACB) [21]. The ACB is associated with reward processing and controlling impulsivity [29–32]. and ACB-projecting hippocampal neurons enhance food palatability in rodents [33]. Further, a recent pilot study in humans revealed that deep brain stimulation in the ACB for patients with severe BED and obesity improved loss-of-control eating frequency and was associated with weight-loss [34]. While previous research on HPC-to-ACB circuitry has focused its role in neuropsychiatric disorders [35, 36], contextual conditioning [37, 38], motivational conflict [39], and non-food related reward processing (e.g., drugs of abuse) [40, 41], we hypothesized that HPC neuronal projections to the ACB, more specifically, ventral HPC (vHPC; field CA1) inputs to the ACB shell subregion (ACBsh), regulate impulsive responding for palatable foods. This hypothesis is evaluated in present experiments in male and female rats in tests of both impulsive action and impulsive choice using complementary functional disconnection and imaging approaches.

## methods and materials

### Animals

Male and female Sprague-Dawley rats (Envigo, Indianapolis, IN; postnatal day [PND] 60-70; 250–275 g on arrival for males, 180–200 g on arrival for females) were individually housed in a temperature-controlled vivarium with *ad libitum* access (except where noted) to water and food (LabDiet 5001, LabDiet, St. Louis, MO) on a 12 h:12 h reverse light/dark cycle. All procedures were approved by the Institute of Animal Care and Use Committee at the University of Southern California and Michigan State University. See Supplementary Fig. 1A for a timeline of all procedures.

### Fiber photometry

In vivo fiber photometry was performed according to previous work [42–45]. Photometry signal was acquired using the Neurophotometrics fiber photometry system (Neurophotometrics, San Diego, CA) at a sampling frequency of 40 Hz and administering alternating wavelengths of 470 nm (Ca^2+^ dependent) or 415 nm (Ca^2+^ independent). All behavioral responses (i.e. lever presses) were time-stamped using the data acquisition software (Bonsai). The resulting signals were then corrected by subtracting the Ca^2+^ independent signal from the Ca^2+^ dependent signal to calculate fluorescence fluctuations due to Ca^2+^ (corrected signal) and not due to baseline neural activity or motion artifacts and fitted to a biexponential curve. The corrected fluorescence signal was then normalized within each rat by calculating the ΔF/F using the average fluorescence signal for the entire recording and converting the signal to *z*-scores. The normalized signal was then aligned to behavioral events via the Bonsai workflow, which interfaces with both the Neurophotometrics systems and the Med Associates system, to extract behavioral timestamps of interest for subsequent peri-event analyses. Data was extracted using the original MATLAB code. To determine the difference in Ca^2+^ dependent activity related to an impulsive vs a non-impulsive response, the area under the curve (AUC) was calculated for the 5-s before and 5-s after a response.

### Differential reinforcement of low rates of responding (DRL)

As previously described [21, 46], minimally food-restricted rats (food removed from home cage 1 h before dark onset) were trained in the early nocturnal phase on an operant lever-pressing task to obtain a single, high-fat, high-sugar 45 mg pellet (HFHS; F05989, Bio-serv; 35% kcal fat and sucrose-enriched) for each press. Training and test sessions were conducted in operant conditioning boxes (Med Associates Inc, St. Albans, VT). After progressively increased delay periods (0-, 5-, 10-second delays, 5 days of training for each schedule) over four weeks of training, rats are given a “DRL-20” schedule for ten days in which they must wait 20 s between lever presses for reinforcement. A lever press prior to the conclusion of the 20-second interval resets the clock, is not reinforced, and is considered an impulsive response. On the other hand, a lever press after the conclusion of the 20-second interval is reinforced and is considered a non-impulsive response. Testing took place on the DRL20 schedule at least 2 days after the completion of training. Efficiency in the task was calculated by taking the ([total amount of reinforced lever presses]/ [total number of active lever presses]) x 100. The periods of 5 s immediately before and after a lever press were determined a priori for analyses of calcium-dependent activity associated with a non-impulsive (reinforced) vs. an impulsive (non-reinforced) press.

### Delay discounting procedure (DD)

As previously described [21], minimally-food restricted rats (food removed from home cage 1 hr before dark onset) were trained in the early nocturnal phase on an operant lever-pressing task to obtain a single, high-fat high-sugar 45 mg pellet (HFHS; F05989, Bio-serv; 35% kcal fat and sucrose-enriched) for each press. Rats were trained in a series of 4 blocks of 5 choice trials (delay training), wherein one lever consistently delivers one pellet and the other delivers 4 pellets. Blocks consisted of a time delay of 0 s, 5 s, 10 s, and 20 s on the delayed lever, with blocks always occurring in that order (i.e. 0 s, 5 s, 10 s, and 20 s delays corresponding to blocks 1, 2, 3, and 4). Each block began with a forced trial for both levers, in random order, during which a stimulus light above each lever was lit, before continuing to the choice trials. During choice trials levers remained extended for 10 s or until a lever response is made, after which the levers were retracted, and the stimulus light remained illuminated until the delivery of the pellet. Training and test sessions were conducted in operant conditioning boxes (Med Associates Inc, St. Albans, VT). Delay training lasted for a total of 24 days, after which testing took place at least 2 days after training. Delay responses were calculated by taking the (# of delay lever responses in completed trials)/(# of total lever responses in completed trials) x 100.

### Meal pattern analysis

Rats were individually housed in a BioDAQ Food and Water intake monitoring system (Research Diets Inc., New Brunswick, NJ), which recorded episodic ad libitum food intake activity from rats in their home cages. All peripheral sensor controllers (PSCs; 1 per cage) were validated for accuracy using 10.00 g standard weights with an allowable margin of error +/– 0.05 g prior to the start of the experiment, as well as before test days. All PSCs were required to have QUIET readings (i.e., no movement at the food hopper) prior to the start of any session. BioDAQ data was analyzed using an inter-meal interval (IMI) of 900 s to separate meals, and bouts were defined as a change in the weight of the PSC that was between –9.00 and 9.00 g in size. Filtering bouts between −9.00 and 9.00 g restricts analyzed events to changes in hopper weight within this range, with positive values reflecting food consumption and negative values arising from brief increases in hopper weight due to animal interaction or system noise. Data was also set to have a period that started at the onset of the dark cycle when the behavioral task would begin, in this case 11:00AM to coincide with the light schedule in the room in which they were housed. The number of periods in the day was set to 24, resulting in 1 h-long period bins from which cumulative intake, as well as average meal size and duration, could be calculated. Following exportation to Microsoft Excel, values were obtained from the “PSC by period” tab of the Excel sheet, giving each sensor's recorded activity broken down into 1 hr periods, to record individual animals’ intake.

### Progressive ratio task (PR)

Rats were trained to lever press for a 45 mg pellet (HFHS; F05989, Bio-serv; 35% kcal fat and sucrose-enriched) in operant conditioning boxes over the course of 6 days, with a 1 h session each day. The first 2 days consisted of a fixed ratio 1 with autoshaping, wherein animals would receive 1 pellet for each correct lever press (FR1), and a pellet would dispense automatically every 10 min. The next 4 days consisted of FR1 without autoshaping (2 days) and FR3 (2 days), wherein animals would receive 1 pellet for every 3 presses on the active lever. On the test day, rats were placed back in the operant chambers to lever press for HFHS pellets under a progressive ratio reinforcement schedule. The response requirement increased progressively using the following formula: F(i) = 5eˆ0.2i-5, where F(i) is the number of lever presses required for the next pellet at i = pellet number and the breakpoint was defined as the final completed lever press requirement that preceded a 20-min period without earning a reinforcer, as described previously [42, 47].

### Peak interval task (PI)

Following viral surgery using DREADDs, rats were individually housed and tested during the onset of the light cycle. During training and testing for the peak interval procedure, rats were restricted to 90% of their baseline weight by providing limited access to a single daily meal of lab chow on completion of training. Behavioral training and testing were similar to previous work [21], and included an initial 15-minute preexposure session with 20% sucrose (w/v) provided in the rats’ home cages, followed by magazine training that was carried out in seven standard operant boxes contained within sound-attenuating cabinets (Med Associates). In these two magazine training sessions, 16 separate presentations of 0.1 ml sucrose were available on a random time 240 s reinforcement schedule. Rats were required to spend ≥10 s in the food magazine during sucrose delivery to move forward to lever training. During lever training, rats received 50 trials, each beginning with the extension of the lever and illumination of the house light. Lever responses after 20 s (Fixed-interval 20; FI 20) led to sucrose delivery, lever retraction and termination of the house light. Following 10 FI 20 sessions, rats received 16 Peak Interval (PI) training sessions. In these sessions, rats received 25 FI trials and 25 probe trials. During these latter trials, their onset was indicated by the illumination of the house light and presentation of the lever; however, no lever presses were reinforced, and the lever was extended through at least 3x the length of the criterion time (i.e., 60 s). Five minutes prior to the test, half the rats received an intraperitoneal injection of either vehicle (1% DMSO in 0.9% saline) or DCZ (100 µg/kg, 1 mL/kg). Testing was identical to the previous sessions, and rats received a three-day interval (washout) with lever and peak interval training. In the final test, rats received the opposite drug contingency to their initial test; thus, all rats received one vehicle and DCZ test. Overall response rates were separated into 1-s bins and then analyzed across each 60-second trial and subsequently averaged across all trials. These response rate data were normalized for each animal based on its maximum response rate during the PI trials.

### Zero Maze task (ZM)

Following established procedures [44], the zero maze apparatus was used to examine exploratory and anxiety-like behavior. The apparatus consisted of an elevated circular track (11.4 cm wide track, 73.7 cm height from track to ground, 92.7 cm exterior diameter) that is divided into four equal-length segments: two sections with 3-cm high curbs (open) and two sections with 17.5-cm height walls (closed). Ambient lighting was used during testing. Rats were placed in the maze on an open section of the track and allowed to roam freely for 5 min. The apparatus was cleaned with 10% ethanol between rats. The total distance traveled and time spent in the open segments of the apparatus were measured via video recording using ANY-maze activity tracking software.

### Statistical analyses

All statistical analyses were conducted using GraphPad Prism 10.0.3 (GraphPad Software, Inc., Boston, Massachusetts, USA). Data are reported as mean ± SEM. T-tests were used to analyze AUC deltas, active lever presses, rewards earned, inactive lever presses, and efficiency in DRL, 1- and 6 h BioDAQ measures (i.e. intake, meal number, meal size) in males and females respectively, breakpoint, active lever presses, and rewards earned in PR, magazine entries/min in the PI task, and distance traveled, time and entries in the open arm in ZM. Two-way ANOVA was used to analyze DD, and a mixed-effects analysis was used to assess differences in BioDAQ measures as a repeated measure from 1 to 6 h in males. A one-way ANOVA was used to assess differences in BioDAQ measures at the 6-h timepoint in females when broken down by estrus cycle. A two-way ANOVA was used to analyze response rate and normalized responses in the PI task. Statistical significance was set at a *p*-value ≤ 0.05.

### Supplemental methods

For methods for estrus tracking, surgical procedures, viral injections, immunohistochemistry, histology, and cell quantification, please see the Supplemental Methods.

## Results

### Calcium-dependent activity in the CA1v predicts control of impulsive action in males

**Fig. 1:**
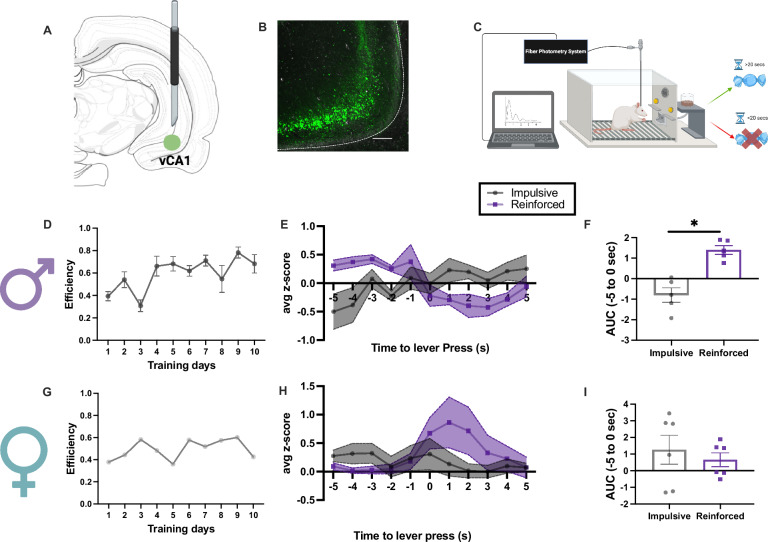
Calcium-dependent activity is increased in both the vHPC and ACBsh prior to a non-impulsive response in the DRL task. **A** Diagram depicting surgical procedure for fiber photometry recordings of Ca^2+^-dependent activity in vHPC. **B** Representative images of surgical targets for the vHPC (Scale bar, 200 um). **C** Diagram depicting the DRL task with photometry recording. **D–F** Ca^2+^-dependent activity is elevated in the 5 s before a non-impulsive lever press, relative to an impulsive press in the vHPC in males (*n* = 5, paired two-tailed t-test, df = 4, *p* = 0.0138) but not (**G–I**) in females (*n* = 6, paired two-tail t-test, df = 5, *p* = 0.4143). (Data are means ± SEM; *p < 0.05).

To examine how CA1v activity relates to impulsive responding for palatable foods, bulk calcium-dependent activity was recorded from the CA1v during the differential reinforcement of low rates of responding (DRL) task in both males and females, as described above (Fig. 1A). Successful viral transfusion and cannula placement were confirmed via histology (Fig. 1B; Supplementary Fig. 1B). Males and females both successfully learned DRL (Fig. 1C), with males averaging 69% efficiency in the task over the final five days of training (Fig. 1D) and females averaging 54% efficiency in the task over the final five days (Fig. 1G), consistent with our previous work [21]. While females displayed a noticeable drop in efficiency in the final training days of DRL, they nevertheless performed similarly to males in the efficiency prior to testing. This drop in efficiency in females at the end of DRL training potentially indicates that animals were overtrained in the task. During testing, calcium-dependent activity in the CA1v was significantly higher in the 5 s preceding a reinforced (i.e., non-impulsive) response, relative to an impulsive (i.e., non-reinforced) response in males (Fig. 1E, F). However, in females, we failed to find a significant difference in calcium-dependent activity in the CA1v in the 5 s preceding a reinforced vs. an impulsive response (Fig. 1H, I). Additionally, calcium-dependent activity in the vHPC was significantly lowered in the 5 s following a reinforced response relative to an impulsive response in males (Supplementary Fig. 1D), but not in females (Supplementary Fig. 1E). These data support the feasibility that the CA1v is functionally involved in regulating impulsive actions directed towards palatable food reinforcement in males but not females.

### Silencing of nucleus accumbens (shell; ACBsh)-projecting CA1v neurons increases impulsive action in males, but not impulsive choice in either sex

**Fig. 2:**
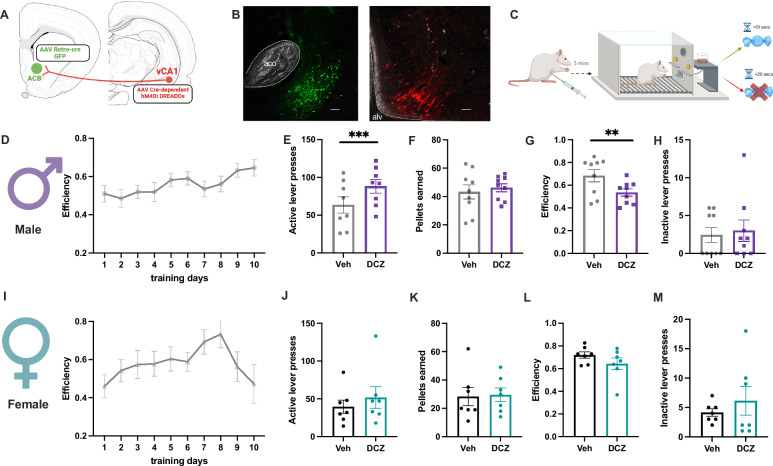
Silencing ACBsh-projecting CA1v neurons increases impulsive action in male rats. **A** Diagram depicting surgical procedure for chemogenetic inactivation of the vHPC-ACBsh pathway. **B** Representative images of surgical targets for the ACBsh (left) and vHPC (right) (Scale bar, 100um). **C** Diagram depicting the DRL task. **D** Training efficiency for males in the DRL task. **E** DREADD inhibition of the vHPC-ACBsh pathway significantly increases active lever presses (*n* = 8, paired two-tail t-test, df = 7, *p* = 0.0005), while **F** number of rewards earned remains unchanged (*n* = 9, paired two-tail t-test, df = 8, *p* = 0.3435). **G** Chemogenetic inhibition of the vHPC-ACBsh pathway significantly decreases efficiency in the DRL task in males (*n* = 9, paired two-tail t-test, df = 8, *p* = 0.0014). **H** Inactive lever presses are unaffected by DCZ administration in the DRL task (*n* = 9, paired two-tail t-test, df = 8, *p* = 0.6172). **I** Training efficiency in females in the DRL task. **J** Active lever presses (*n* = 7, paired two-tail t-test, df = 6, *p* = 0.1180) **K** rewards earned (*n* = 7, paired two-tail t-test, df = 6, *p* = 0.6661), **L** efficiency (*n* = 7, paired two-tail t-test, df = 6, *p* = 0.2077), and **M** inactive lever presses (*n* = 7, paired two-tail t-test, df = 6, *p* = 0.3216) are unaffected by chemogenetic inhibition of the vHPC-ACBsh pathway in females. (Data are means ± SEM; **p < 0.01, ***p < 0.001).

Based on previous work associating the ACB with impulse control [30, 31], and the fact that CA1v neurons project directly to the ACBsh [21]. We assessed whether CA1v projections to the ACBsh impact impulsivity in DRL. Animals were injected with a retrograde cre-expressing virus in the ACBsh and a cre-dependent inhibitory DREADDs in the CA1v, an approach that selectively targets CA1v neurons that project to the ACBsh for reversible chemogenetic inhibition (Fig. 2A). Successful viral transfection was confirmed via immunohistochemistry (Fig. 2B). Animals were trained in the DRL task of impulsive action (Fig. 2C). Animals successfully learned the task, averaging 59% (males) and 61% (females) efficiency in the task over the final five days of training (Fig. 2D, I). On test days, animals received intraperitoneal (IP) injections of either Deschloroclozapine (DCZ; DREADDs ligand) or vehicle to selectively inhibit ACBsh-projecting CA1v neurons. Results revealed that males receiving DCZ made significantly more responses on the active lever relative to animals receiving vehicle, while the number of rewards earned was unaffected (Fig. 2E, F), resulting in decreased efficiency in males receiving DCZ relative to vehicle (i.e., increased impulsive action; Fig. 2G). There were no changes with inactive lever presses with DCZ administration in males, suggesting that overall motor impulsivity is unaffected by chemogenetic silencing of ACBsh-projecting CA1v neurons (Fig. 2H). However, females showed no change in task efficiency, lever presses, inactive lever presses, or rewards earned following DCZ treatment relative to vehicle (Fig. 2J–M), even when controlling for the effects of estrus cycle stage (Supplementary Fig. 2A–D).

As a control to ensure that DCZ administration itself was not playing a role in impulsive responding, a separate group of male rats was injected with cre-dependent inhibitory DREADDs in the CA1v, but were not given a retrograde, cre-expressing virus in the ACBsh, rendering the DREADDs nonfunctional. Administration of DCZ prior to DRL had no effect on efficiency, total lever presses, inactive lever presses, or rewards earned in this group (Supplementary Fig. 2I–L).

While the full neural circuitry regulating impulsive responding is poorly understood, evidence suggests that impulsive actions and impulsive choices are modulated by both overlapping and distinct neural substrates [15, 21]. DRL is an operant test for impulsive actions, but fails to address questions of impulsive choices. Therefore, to test whether inhibition of the CA1v-to-ACBsh pathway affects impulsive choices, we paired our viral approach described above with a delay discounting (DD) task (Supplementary Fig. 2I). Delay discounting refers to the tendency to devalue a reward as the time delay associated with delivery of the reward increases. During testing, animals of both sexes exhibited standard discounting behavior, as exhibited by decreased responding on the delayed lever as the time delay on the larger reward lever progressively increased. However, no overall differences were found between DCZ- and vehicle-treated animals in males (Supplementary Fig. 2J) or females (Supplementary Fig. 2K, L), indicating that the CA1v-to-ACBsh pathway does not regulate impulsive choice (Supplementary Fig. 2J, K). Taken together, these results show that reversible inhibition of ACBsh-projecting CA1v neurons affects impulsivity in a sex- and task-dependent manner, thus further emphasizing the importance of evaluating both sex and behavioral procedure as variables for research on impulsivity.

### Silencing of ACBsh-projecting CA1v neurons does not affect peak interval timing, food intake, motivation to work for palatable food, or anxiety-like behavior

**Fig. 3:**
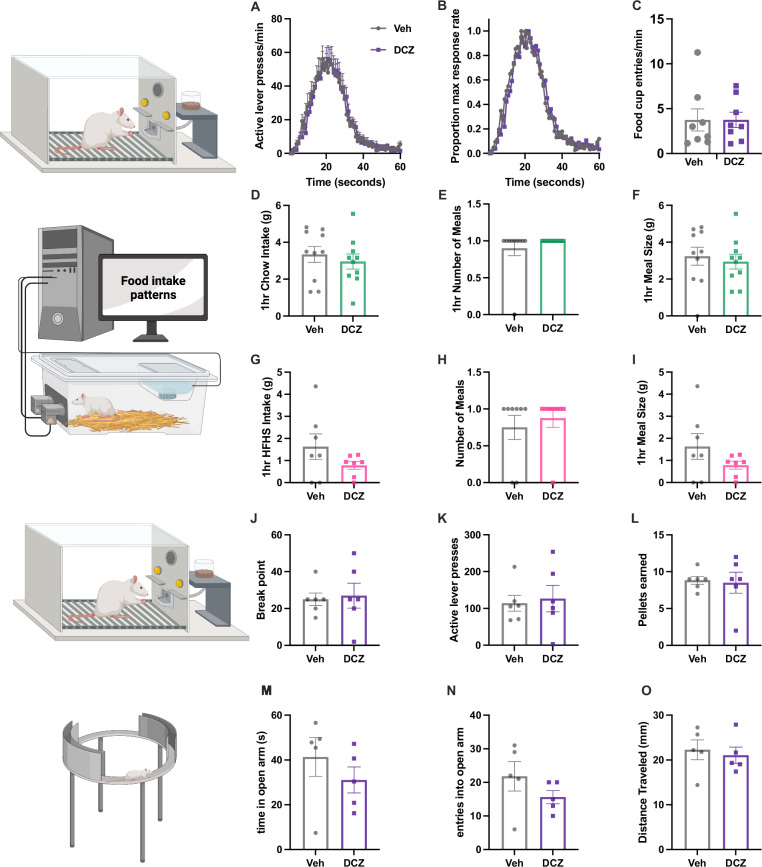
Silencing ACBsh-projecting CA1v neurons does not affect peak interval timing, home cage intake of standard chow, HFHS chow, reward motivation, or anxiety-like behavior. Peak interval timing was unaffected by chemogenetic inhibition of CA1v-ACBsh pathway neurons, with ANOVA only showing a main effect of time for (**A**), response rate (F(59, 826) = 44.41, *p* < 0.001) and **B** normalized responses (F(59,826) = 71.72, *p* < 0.001). **C** Magazine entries/min showed no significant differences (*n* = 8, paired two-tail t-test, df = 7, *p* = 0.97). **D** Total chow intake (*n* = 10, paired two-tail t-test, df = 9, *p* = 0.6315) **E** number of meals (*n* = 10, paired two-tail t-test, df = 9, *p* = 0.3434) and **F** average meal size (*n* = 10, paired two-tailed t-test, df = 9, *p* = 0.7358) were unaffected by chemogenetic silencing at the 1 h timepoint for standard chow. Likewise, **G** Total chow intake (*n* = 7, paired two-tail t-test, df = 6, *p* = 0.1770), **H** number of meals (*n* = 8, paired two-tail t-test, df =7 6, *p* = 0.5983), and **I** average meal size (*n* = 7, paired two-tail t-test, df = 6, *p* = 0.1760) were also unaffected by chemogenetic silencing at the 1 h timepoint for HFHS chow. No differences were seen in reward motivation, as measured in the PR task, with (**J**) breakpoint (*n* = 6, paired two-tail t-test, df = 5, *p* = 0.6512), **K** active lever presses (*n* = 6, paired two-tail t-test, df = 5, *p* = 0.5998), and **L** rewards earned (*n* = 6, paired two-tail t-test, df = 5, *p* = 0.7575) all unaffected by chemogenetic inhibition of the CA1v-ACB pathway. Finally, **M** time spent in the open arm (*n* = 5, unpaired two-tail t-test, df = 8, *p* =0.3554), **N** number of entries into the open arm (*n* = 5, unpaired two-tail t-test, df = 8, *p* = 0.2340), and **O** total distance traveled (*n* = 5, unpaired two-tail t-test, df = 8, *p* =0.6849) were not significantly different in the zero maze task (within-subjects for peak interval, food intake and reward motivation, between-subjects for anxiety-like behavior; Data are means ± SEM; *p < 0.05, ***p < 0.001).

To test whether our results might be influenced by behavioral changes other than impulsivity, we performed a variety of behavioral assays to determine the effects of reversible inhibition of the CA1v-ACBsh pathway on peak interval timing (an established test of timing capacity in rats [21], spontaneous food intake (and meal patterns), food reward motivation, and anxiety-like behavior. Given that our phenotype for impulsivity was not evident in females, these experiments were performed in male rats. DCZ treatment did not impact performance during peak interval testing. Both overall response rate (Fig. 3A) and the normalized proportion of peak rate responding (Fig. 3B) were comparable under vehicle and DCZ conditions. Similarly, magazine entries/min were comparable between vehicle and DCZ conditions (Fig. 3C). Overall, these findings indicate comparable interval timing of the 20 s criterion duration irrespective of chemogenetic inactivation of the CA1v-ACBsh pathway, suggesting that disruption of this pathway in male rats promotes impulsive responding despite intact timing capacity.

Inhibition of the CA1v-ACBsh pathway also did not affect cumulative intake of standard chow (Fig. 3D), nor did it affect the number of meals eaten (Fig. 3E) or the average meal size (Fig. 3F) for males at the 1 hr timepoint (i.e., the time at which DCZ would affect would be most robust). Additionally, the meal parameters listed above were also unaffected by DCZ administration over a 2 h, 3 h, 5 h, or 6 h period (Supplementary Fig. 3A–C) in males. Inhibition of this pathway also failed to affect these same parameters when males were given a high-fat, high-sugar (HFHS) chow (Fig. 3G–I; Supplementary Fig. 3D–F). Finally, we saw no effect of CA1v-ACBsh pathway inhibition on these same meal parameters in females at the 1 hr time point (Supplementary Fig. G–I) or when taking estrus stage into account (Supplementary Fig. J–L). Overall reward motivation was not affected by the CA1v-ACBsh pathway inhibition in the progressive ratio task in male rats (Fig. 3J–L), nor was anxiety-like or exploratory behavior in the zero maze test (Fig. 3M–O).

### Increased impulsive action in males is specific to CA1v-ACBsh synaptic communication

**Fig. 4:**
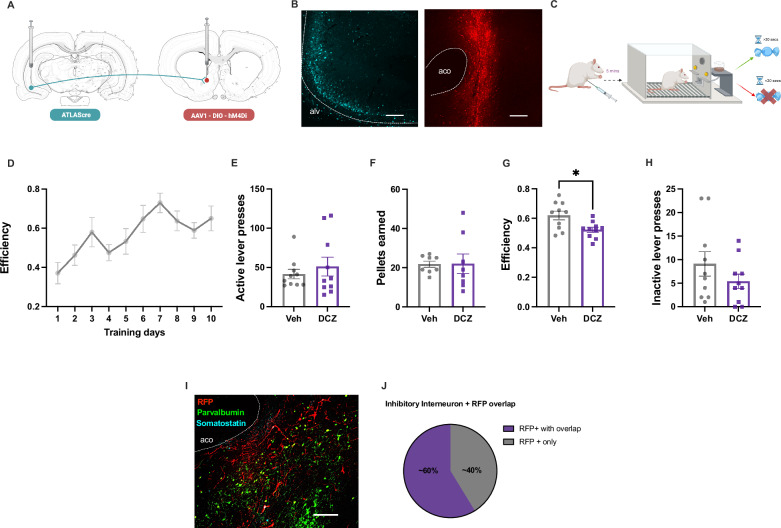
Increased impulsive action in males is specific to CA1v to ACB signaling. **A** Diagram depicting surgical procedure for chemogenetic inhibition of ACB neurons which receive projections from the CA1v. **B** Representative images of surgical targets for the ACBsh (left) and vHPC (right) (Scale bar, 100um). **C** Diagram depicting the DRL task. **D** Training efficiency for the DRL task. **E** Active lever presses (*n* = 10, paired two-tail t-test, df = 9, *p* = 0.4986), **F** rewards earned (*n* = 8, paired two-tail t-test, df = 7, *p* = 0.9566), **G** task efficiency (*n* = 10, paired two-tail t-test, df = 9, *p* = 0.0421) and **H** inactive lever presses (*n* = 10, paired two-tail t-test, df = 9, *p* = 0.1407) with chemogenetic inhibition of ACB neurons receiving projections from the CA1v in males. **I, J** A majority of ACB neurons receiving projections from the CA1v are inhibitory interneurons (*n* = 3) (Data are means ± SEM; *p < 0.05).

Given that ventral HPC CA1 neurons are known to have collateral projections to various hypothalamic, striatal, septal, and cortical targets [42, 45], we tested whether our results might be driven by collateral projections from ACBsh-projecting CA1v neurons, vs direct CA1v->ACBsh signaling. We utilized a novel viral approach developed to target downstream neurons in a strictly anterograde, monosynaptic, and glutamatergic synaptic activity-dependent manner (ATLAS) [48]. We paired this novel transsynaptic viral approach in the CA1v—in this case to drive cre recombinase expression downstream of CA1v glutamatergic terminals—with a cre-dependent inhibitory DREADD in the ACBsh, to effectively isolate the CA1v-ACBsh-specific pathway for reversible silencing (Fig. 4A, B). Animals were then trained in DRL (Fig. 4C) and successfully learned the task, averaging 57% efficiency in the task over the final five days of training (Fig. 4D). Results reveal that animals receiving DCZ made more presses on the active lever (Fig. 4E), while earning a similar number of rewards (Fig. 4F) compared to animals receiving vehicle. While this difference in active lever presses was not statistically significant, it nevertheless contributed to a significant decrease in efficiency in the DRL task when animals received DCZ relative to vehicle (Fig. 4G). There were no changes with inactive lever presses with DCZ administration, suggesting that overall motor impulsivity is unaffected by chemogenetic silencing of ACBsh neurons which receive projections from the CA1v (Fig. 4H). These results indicate that our earlier findings revealing that chemogenetic inhibition of ACBsh-projecting CA1v neurons increases impulsivity in this task were not due to inhibition of collateral projections of these neurons, but rather, were driven by inhibition of Ca1v to ACBsh glutamatergic signaling.

### CA1v neurons target inhibitory interneurons in the ACBsh

To gain insight into the phenotype of ACBsh neurons receiving projections from the CA1v, we performed IHC to co-stain for ACBsh neurons that receive synaptic input from CA1v neurons (a neuron which has a glutamatergic synaptic connection based on ATLAS viral transmission; RFP+ neurons) and the inhibitory interneuron markers, parvalbumin and somatostatin. Results show that roughly 60% of the RFP+ neurons in the ACBsh (i.e. neurons which receive projections from the CA1v) are inhibitory interneurons (Fig. 4I, J), expressing either somatostatin or parvalbumin. These results indicate that HPCv->ACBsh regulation of impulsivity in males may predominantly involve modulation of intrinsic ACBsh activity via interneuron inhibitory GABAergic signaling.

## Discussion

Food-directed impulsivity is strongly implicated in obesity, gambling, and binge-eating disorder [1, 2, 5, 6], and these associations may be driven by neural circuits that connect the hippocampus (HPC) with the nucleus accumbens (ACB) [34, 49, 50]. Present results reveal that calcium-dependent activity in the ventral HPC (vHPC; CA1v region) is significantly elevated prior to a non-impulsive lever press relative to an impulsive press in the operant DRL test of impulsive action in males but not in females, suggesting that the CA1V region may play a functional role in regulating impulse control in a sex-specific manner. Given the direct downstream projections from CA1v neurons to the ACBsh [21], as well as the previous work associating the ACBsh with impulse control [31], we explored the role of the CA1v-ACBsh pathway in regulating impulsive responding for palatable food. Consistent with these findings, chemogenetic silencing of CA1v neurons that project to the ACBsh resulted in significantly decreased efficiency in DRL, an outcome indicative of increased impulsive action. However, this result was only seen in males as chemogenetic inhibition of the CA1v-ACBsh pathway in females did not impact DRL performance, regardless of estrus stage. Furthermore, we found that chemogenetic inhibition of the CA1v-ACB pathway did not affect impulsive choice, as measured using the delay discounting task, in either male or females, nor did it affect home cage consumption of standard or HFD chow, motivation to work for palatable food, or anxiety-like behavior in males. Importantly, we also found no differences in peak interval timing with chemogenetic inhibition of ACBsh-projecting CA1v neurons, suggesting that differences seen in DRL performance in males are not caused by changes in timing capacity performance. By combining a novel transsynaptic glutamatergic synapse-based ATLAS approach [48] with chemogenetic silencing and immunostaining, we reveal that the CA1v-ACBsh pathway’s control of impulsive behavior is indeed pathway-specific to CA1v synapses on ACBsh neurons and not collateral targets of these neurons. Additionally, we find that CA1v neurons communicate predominantly to inhibitory interneurons within the ACBsh.

Our findings that activity in the vHPC is significantly elevated in the 5 s prior to a non-impulsive response relative to an impulsive one in the DRL task in males is in line with previous research, which suggests a role for activity in the CA1v in regulating behavioral inhibition [29, 51]. The significant difference in calcium-dependent activity in the 5 s following a non-impulsive response relative to an impulsive one in males further suggests that activity in the CA1v is dynamically altered based either on impulse control (or lack thereof) prior to the response, and/or reinforcement delivery, as non-impulsive vs. impulsive responses differ based on both of these variables. It should be noted that the genetically encoded calcium indicator used in this study was driven by an hSYN promoter, meaning viral expression would be transduced in all neurons in the brain region in which it was infused. While a virus driven by a more specific promoter, such as CAMKIIA for CA1v infusions, would increase the specificity of these experiments, pathway specificity was confirmed in the functional studies.

Both the vHPC and ACB have recently emerged as likely substrates in regulating impulsive responding [21, 22, 29–32], and thus we sought to investigate the role of the CA1v-ACBsh pathway in mediating multiple forms of impulse control, looking at both impulsive actions and impulsive choices. Previous research suggests impulsive action and impulsive choice are dissociable behaviors [15, 52–54]. with studies finding that outcomes in impulsive choice and action measures are not correlated in either humans or rats [16]. The vHPC has previously been implicated in the regulation of both impulsive actions [21, 47, 51] and impulsive choices [55–58], with vHPC loss-of-function approaches leading to increases in impulsive responding. While our results, revealing that vHPC-ACBsh pathway inhibition increases impulsive actions, are consistent with previous literature, our lack of effect on impulsive choice warrants follow-up consideration. Given the pathway-specific nature of the current study, which uses two distinct but complementary disconnection approaches, it is difficult to draw direct comparisons to previous research that targeted this pathway with less specificity. For example, Abela et al. investigated the role of the vHPC-ACBsh pathway in regulating impulsive choices using an excitotoxic contra-lesional approach, finding a significant difference in delay discounting behavior in animals with a disconnected vHPC-ACBsh pathway [56]. However, contra-lesional approaches are not pathway-specific and disrupt collateral projection targets to and from both regions. Further, these approaches are chronic and not reversible, and are thus vulnerable to compensatory neural adaptations. Therefore, our lack of an observed effect on impulsive choice behavior is likely based on the increased sensitivity of our pathway-specific manipulations compared to previous lesion-based literature. Additionally, previous research shows dissociable effects of impulsive responding with targeting of the ACB core (ACBc) vs the ACB shell (ACBsh) [59]. For example, Sesia et al. found that deep brain stimulation of the ACBsh increased impulsive action, while DBS of the ACBc did not [59]. Taken together with previous literature, our results indicate that the influence of vHPC signaling on impulsive choice may be based on projections to targets other than the ACBsh (e.g., lateral hypothalamus or medial prefrontal cortex).

While we found that inhibition of ACBsh-projecting CA1v neurons increased impulsive action in males, we found no effect on either impulsive action or impulsive choice in females. Research investigating sex differences in impulsive responding is mixed, with outcomes in humans varying depending on the population and task being studied [19, 20, 60]. In animal models, outcomes can vary based on the developmental timing of testing [17, 18, 20, 61], though males tend to exhibit higher impulsivity in impulsive actions [18, 20, 62, 63], whereas females are more prone to impulsive choices [20]. Our current results are largely consistent with these findings. However, previous studies have shown a role of estrus/menstrual cycle in the regulation of impulsivity in females [64–67]. We failed to find an effect on impulsivity in females, even when controlling for estrus stage. However, Patterson et al. looked specifically at the effects of vHPC-ACBsh inhibition and found subtle sex differences in behavior during motivational conflict [39], suggesting that CA1v-ACBsh inhibition can have differing effects based on sex, consistent with our results.

The vHPC is known to play an important role in regulating food intake control [23–27]. While we saw no effect on cumulative food intake or meal patterns with inhibition of the CA1v-ACBsh pathway, differences between our results and previous findings may be explained by distinct functions of CA1v projection pathways. For example, previous work from our lab reveals that CA1v projections to the lateral hypothalamic area (via ghrelin receptor action) and to the medial prefrontal cortex (via GLP-1 receptor action) potently increase and reduce food intake, respectively [22, 47, 68, 69]. A recent study found that activity of ACB-projecting vHPC neurons increased upon food investigation and that this activity inhibited the transition to meal initiation in mice [70]. However, consistent with our results, chemogenetic manipulation of this pathway in mice did not influence overall food consumption in the long-term [70]. Thus, our current findings that inhibition of the CA1v-ACBsh pathway promotes impulsive actions but not food consumption parallel previous work identifying a role for the CA1v-ACBsh pathway in food anticipatory but not longer-term consummatory behavior, the latter of which is likely driven more by CA1v projections to the lateral hypothalamus and prefrontal cortex.

Considering the significant difference in active lever presses in DRL between control and CA1v->ACB disconnection treatments, we examined whether CA1v-ACB signaling may play a role in willingness to work for palatable food as a readout of food motivation independent from impulsivity. Previous research shows a role for dorsal HPC-ACB circuitry in regulating reward motivation [71, 72], but the role of vHPC-ACB circuitry is less clear. Yang and colleagues showed that the low-frequency photostimulation of vHPC neurons that project to the ACB increases the number of licks per bout—a measure of food palatability—when mice are given access to a sucrose solution, though there was no difference in total bout number—a measure of satiation. Additionally, they revealed that photoinhibition of vHPC neurons that project to the ACB disrupts sucrose-driven flavor preference conditioning [33], indicating a role for this pathway in nutritive-based reinforcement learning. This study highlights a role for CA1v-ACB activity in mediating the early ingestive phase positive hedonic experience, but reveals little about the effects on effort-based motivation to obtain palatable foods. Thus, our findings that inhibition of the CA1v-ACB pathway does not affect operant behavior in the PR task add to existing literature to suggest that CA1v-ACB circuitry is implicated in behaviors associated with early ingestive phase food reward processes (e.g., food reinforcement-based learning, hedonic evaluations) but does not have a direct effect on effort-based operant responding for palatable food.

Given that previous research suggests a role for the HPC in guiding anxiety-like behavior [73, 74], we also investigated the effects of CA1v-ACB inhibition on anxiety-like behavior in the zero maze test. Chemogenetic inhibition of the CA1v-ACB pathway had no effect on anxiety-like behavior as measured in this task, consistent with previous pathway-specific vHPC studies [36, 39, 75]. Bagot and colleagues found no differences with photostimulation of the vHPC-ACB pathway when behavior was assessed using the open field test of anxiety (OFT) [36], whereas Patterson et al. also found no differences in anxiety-like behavior when measured using the OFT or the elevated plus maze when inhibiting the vHPC-ACB pathway [39]. Thus, our results align with previous reports in revealing little-to-no role of vHPC-ACB circuitry specifically in anxiety-like behavior, collectively indicating that vHPC influences on anxiety likely involve other projection targets.

While prior studies show a role for other pathways originating from the vHPC in shaping impulsive behavior [22, 76, 77] our study suggests using a novel transsynaptic glutamatergic synapse targeting approach [48] that the CA1v-ACB specific pathway plays a role independent of possible collateral projection targets. Additionally, we show that a majority of ACB neurons receiving CA1v projections are inhibitory interneurons. Previous research has shown an important role for inhibitory interneurons in modulating ACB activity, and excitatory dorsal HPC (dHPC) neurons that project to ACB medium spiny neurons (MSNs) also target inhibitory interneurons within the ACB. Thus, the present results combined with these previous findings suggest that multiple hippocampal pathways (from both dorsal and ventral subregions) converge on ACB inhibitory interneurons in the ACB to modulate activity in this region. Future studies are required to determine whether vHPC and dHPC inputs to the ACB converge on common vs distinct neural systems to regulate fundamental behaviors.

In addition to obesity and BED, impulsivity has been implicated in a number of neuropsychiatric disorders, such as compulsive gambling [9]. substance use disorders [7, 8, 78], and schizophrenia [79–82]. Similarly, the HPC and ACB have been implicated in many psychiatric conditions, with HPC-ACB circuitry specifically implicated in depression [35, 36, 83]. substance use disorders [84–86]. and schizophrenia [87–91]. The glutamatergic hypothesis of schizophrenia suggests that hypoactivity of inhibitory interneurons in the corticolimbic circuit may play a crucial role in the development of schizophrenia, particularly parvalbumin+ interneurons [91–93], which make up the majority of the interneurons identified in this study. Further, the activity of ACB interneurons affects impulsive action [32]. Future research is needed to investigate the role of ACB-projecting CA1v neurons in schizophrenia pathology, with a focus on whether dysregulation of ACB parvalbumin+ interneurons that receive projections from the Ca1v plays a role in disease etiology.

In summary, our results reveal that activity in the CA1v-ACB pathway plays a sex- and task-dependent role in regulating impulsive responding for palatable foods. Calcium-dependent activity in the CA1v is significantly elevated prior to a non-impulsive lever press relative to an impulsive press in a test of impulsive action in males, suggesting a role for the region in mediating impulse control. Functional testing revealed that chemogenetic inhibition of the Ca1v-ACBsh pathway—using multiple complementary disconnection approaches—increased impulsive action in males yet had no effect on either impulsive actions or impulsive choices in females. These changes in impulsive action in males are not secondary to changes in food intake, willingness to work for palatable food, or anxiety-like behavior, as these measures were unaffected, with pathway inhibition. Future research should further investigate the role of male and female sex hormones in the regulation of this pathway, given the sexual dimorphism observed here. Additionally, characterization of the receptor profiles of both CA1v and ACBsh neurons in this pathway is warranted, as previous research suggests a role for multiple neurotransmitters, including the dopaminergic [30, 94–96], opioidergic [96–99], and melanin-concentrating hormone [21] systems in regulating impulsive responding for palatable foods.

## Supplementary information

Supplemental Information

## Data Availability

All data are available upon request. Please contact the corresponding author for access.

## References

[1] Lavagnino L, Arnone D, Cao B, Soares JC, Selvaraj S. Inhibitory control in obesity and binge eating disorder: a systematic review and meta-analysis of neurocognitive and neuroimaging studies. Neurosci Biobehav Rev. 2016;68:714–26. 27381956 10.1016/j.neubiorev.2016.06.041

[2] Giel K, Teufel M, Junne F, Zipfel S, Schag K. Food-related impulsivity in obesity and binge eating disorder—a systematic update of the evidence. Nutrients. 2017;9:1170. 29077027 10.3390/nu9111170 PMC5707642

[3] Liu Z-N, Jiang J-Y, Cai T-S, Zhang D-L. A study of response inhibition in overweight/obesity people based on event-related potential. Front Psychol. 2022;13:826648. 35310211 10.3389/fpsyg.2022.826648 PMC8929195

[4] Mobbs O, Iglesias K, Golay A, Van der Linden M. Cognitive deficits in obese persons with and without binge eating disorder. Investigation using a mental flexibility task. Appetite. 2011;57:263–71. 21600255 10.1016/j.appet.2011.04.023

[5] Meule A, de Zwaan M, Müller A. Attentional and motor impulsivity interactively predict ‘food addiction’ in obese individuals. Compr Psychiatry. 2017;72:83–87. 27768944 10.1016/j.comppsych.2016.10.001

[6] VanderBroek-Stice L, Stojek MK, Beach SRH, vanDellen MR, MacKillop J. Multidimensional assessment of impulsivity in relation to obesity and food addiction. Appetite. 2017;112:59–68. 28087369 10.1016/j.appet.2017.01.009 PMC5344714

[7] Dezhkam, N, Zarbakhsh Bahri, MR & Khaneh Keshi, A. The effect of impulsivity on addiction and addictive tendencies: a meta-analysis. J Health Rep Technol. 2022;8e120846.

[8] Lee RSC, Hoppenbrouwers S, Franken I. A systematic meta-review of impulsivity and compulsivity in addictive behaviors. Neuropsychol Rev. 2019;29:14–26. 30927147 10.1007/s11065-019-09402-x

[9] Chowdhury NS, Livesey EJ, Blaszczynski A, Harris JA. Pathological gambling and motor impulsivity: a systematic review with meta-analysis. J Gambl Stud. 2017;33:1213–39. 28255940 10.1007/s10899-017-9683-5

[10] Emmerich S, Fryar C, Stierman B, Ogden, C. Obesity and severe obesity prevalence in adults: United States. 2023. https://stacks.cdc.gov/view/cdc/159281 (2024) 10.15620/cdc/159281. 10.15620/cdc/159281 PMC11744423 39808758

[11] Volkow ND, Wang G-J, Baler RD. Reward, dopamine and the control of food intake: implications for obesity. Trends Cogn Sci. 2011;15:37–46. 21109477 10.1016/j.tics.2010.11.001 PMC3124340

[12] Maxwell AL, Gardiner E, Loxton NJ. Investigating the relationship between reward sensitivity, impulsivity, and food addiction: a systematic review. Eur Eat Disord Rev. 2020;28:368–84. 32142199 10.1002/erv.2732

[13] Morys F et al. Neuroanatomical correlates of genetic risk for obesity in children. Transl Psychiatry. 2023;13:1. 10.1038/s41398-022-02301-5 PMC9810659 36596778

[14] García-García I, et al. Relationship between impulsivity, uncontrolled eating and body mass index: a hierarchical model. Int J Obes. 2022;46:129–36. 10.1038/s41366-021-00966-4 34552208

[15] Wang Q, et al. Dissociated neural substrates underlying impulsive choice and impulsive action. NeuroImage. 2016;134:540–9. 27083527 10.1016/j.neuroimage.2016.04.010

[16] Broos N, et al. The relationship between impulsive choice and impulsive action: a cross-species translational study. PLoS ONE. 2012;7:e36781. 22574225 10.1371/journal.pone.0036781 PMC3344935

[17] Burton CL, Fletcher PJ. Age and sex differences in impulsive action in rats: The role of dopamine and glutamate. Behav Brain Res. 2012;230:21–33. 22326372 10.1016/j.bbr.2012.01.046

[18] Bayless DW, Darling JS, Stout WJ, Daniel JM. Sex differences in attentional processes in adult rats as measured by performance on the 5-choice serial reaction time task. Behav Brain Res. 2012;235:48–54. 22835820 10.1016/j.bbr.2012.07.028

[19] Gaillard A, Fehring DJ, Rossell SL. A systematic review and meta-analysis of behavioural sex differences in executive control. Eur J Neurosci. 2021;53:519–42. 32844505 10.1111/ejn.14946

[20] Weafer J, de Wit H. Sex differences in impulsive action and impulsive choice. Addict Behav. 2014;39:1573–9. 24286704 10.1016/j.addbeh.2013.10.033 PMC4012004

[21] Noble EE, et al. Hypothalamus-hippocampus circuitry regulates impulsivity via melanin-concentrating hormone. Nat Commun. 2019;10:4923. 31664021 10.1038/s41467-019-12895-y PMC6820566

[22] Hsu TM et al. A hippocampus to prefrontal cortex neural pathway inhibits food motivation through glucagon-like peptide-1 signaling. Mol Psychiatry. 2018;23:1555–65. 28461695 10.1038/mp.2017.91 PMC5668211

[23] Stevenson RJ, Francis HM. The hippocampus and the regulation of human food intake. Psychol Bull. 2017;143:1011–32. 28616995 10.1037/bul0000109

[24] Higgs S. Memory and its role in appetite regulation. Physiol Behav. 2005;85:67–72. 15924907 10.1016/j.physbeh.2005.04.003

[25] Clasen MM, Riley AL, Davidson TL. Hippocampal-dependent inhibitory learning and memory processes in the control of eating and drug taking. CPD. 2020;26:2334–52. 10.2174/1381612826666200206091447 32026771

[26] Hannapel R, et al. Postmeal optogenetic inhibition of dorsal or ventral hippocampal pyramidal neurons increases future intake. eNeuro. 2019;6:ENEURO.0457-18.2018. 30693314 10.1523/ENEURO.0457-18.2018 PMC6348449

[27] Hannapel RC, et al. Ventral hippocampal neurons inhibit postprandial energy intake. Hippocampus. 2017;27:274–84. 28121049 10.1002/hipo.22692

[28] Barbosa DAN, et al. An orexigenic subnetwork within the human hippocampus. Nature. 2023;621:381–8. 37648849 10.1038/s41586-023-06459-w PMC10499606

[29] Basar K, et al. Nucleus accumbens and impulsivity. Prog Neurobiol. 2010;92:533–57. 20831892 10.1016/j.pneurobio.2010.08.007

[30] Besson M, et al. Dissociable control of impulsivity in rats by dopamine d2/3 receptors in the core and shell subregions of the nucleus accumbens. Neuropsychopharmacol. 2010;35:560–9. 10.1038/npp.2009.162 PMC3055378 19847161

[31] Feja M, Hayn L, Koch M. Nucleus accumbens core and shell inactivation differentially affects impulsive behaviours in rats. Prog Neuro-Psychopharmacol Biol Psychiatry. 2014;54:31–42. 10.1016/j.pnpbp.2014.04.012 24810333

[32] Pisansky MT, et al. Nucleus accumbens fast-spiking interneurons constrain impulsive action. Biol Psychiatry. 2019;86:836–47. 31471038 10.1016/j.biopsych.2019.07.002 PMC6823148

[33] Yang AK, Mendoza JA, Lafferty CK, Lacroix F, Britt JP. Hippocampal input to the nucleus accumbens shell enhances food palatability. Biol Psychiatry. 2020;87:597–608. 31699294 10.1016/j.biopsych.2019.09.007

[34] Shivacharan, RS et al. Pilot study of responsive nucleus accumbens deep brain stimulation for loss-of-control eating. Nat Med. 2022. 10.1038/s41591-022-01941-w. 10.1038/s41591-022-01941-w PMC9499853 36038628

[35] Medrihan, L et al. Glutamatergic projections from ventral hippocampus to nucleus accumbens cholinergic neurons are altered in a mouse model of depression. 2022 10.1101/2022.04.20.488950. 10.1085/jgp.202413693 PMC11893161 40052940

[36] Bagot RC, et al. Ventral hippocampal afferents to the nucleus accumbens regulate susceptibility to depression. Nat Commun. 2015;6:7062. 25952660 10.1038/ncomms8062 PMC4430111

[37] Ito R, Robbins TW, Pennartz CM, Everitt BJ. Functional interaction between the hippocampus and nucleus accumbens shell is necessary for the acquisition of appetitive spatial context conditioning. J Neurosci. 2008;28:6950–9. 18596169 10.1523/JNEUROSCI.1615-08.2008 PMC3844800

[38] LeGates TA, et al. Reward behaviour is regulated by the strength of hippocampus–nucleus accumbens synapses. Nature. 2018;564:258–62. 30478293 10.1038/s41586-018-0740-8 PMC6292781

[39] Patterson D, et al. Ventral hippocampus to nucleus accumbens shell circuit regulates approach decisions during motivational conflict. PLOS Biol. 2025;23:e3002722. 39854559 10.1371/journal.pbio.3002722 PMC11761569

[40] Zhou Y, et al. A ventral CA1 to nucleus accumbens core engram circuit mediates conditioned place preference for cocaine. Nat Neurosci. 2019;22:1986–99. 31719672 10.1038/s41593-019-0524-y

[41] Zhou Y, et al. The projection from ventral ca1, not prefrontal cortex, to nucleus accumbens core mediates recent memory retrieval of cocaine-conditioned place preference. Front Behav Neurosci. 2020;14:558074. 33304246 10.3389/fnbeh.2020.558074 PMC7701212

[42] Décarie-Spain L et al. Ventral hippocampus-lateral septum circuitry promotes foraging-related memory. Cell Rep. 2022;40:111402. 10.1016/j.celrep.2022.111402 PMC9605732 36170832

[43] Subramanian KS, et al. Hypothalamic melanin-concentrating hormone neurons integrate food-motivated appetitive and consummatory processes in rats. Nat Commun. 2023;14:1755. 36990984 10.1038/s41467-023-37344-9 PMC10060386

[44] Hayes AMR, et al. Western diet consumption impairs memory function via dysregulated hippocampus acetylcholine signaling. Brain Behav Immun. 2024;118:408–22. 38461956 10.1016/j.bbi.2024.03.015 PMC11033683

[45] Décarie-Spain L, et al. Ventral hippocampus neurons encode meal-related memory. Nat Commun. 2025;16:4898. 40500290 10.1038/s41467-025-59687-1 PMC12159138

[46] Klug ME, et al. Endotoxemia-induced inflammation in the absence of obesity is associated with decreased anxiety-like and impulsive behavior with no effect on learning and memory. Compr Physiol. 2025;15:e70044. 40884034 10.1002/cph4.70044 PMC12397682

[47] Hsu TM, Hahn JD, Konanur VR, Lam A, Kanoski SE. Hippocampal GLP-1 receptors influence food intake, meal size, and effort-based responding for food through volume transmission. Neuropsychopharmacol. 2015;40:327–37. 10.1038/npp.2014.175 PMC4443945 25035078

[48] Rivera JF, et al. ATLAS: a rationally designed anterograde transsynaptic tracer. Nat Methods. 2025;22:1101–11. 40312509 10.1038/s41592-025-02670-x PMC12074993

[49] Rolle CE, et al. Accumbens connectivity during deep-brain stimulation differentiates loss of control from physiologic behavioral states. Brain Stimulation. 2023;16:1384–91. 37734587 10.1016/j.brs.2023.09.010 PMC10811591

[50] Kanoski SE, Davidson TL. Western diet consumption and cognitive impairment: links to hippocampal dysfunction and obesity. Physiol Behav. 2011;103:59–68. 21167850 10.1016/j.physbeh.2010.12.003 PMC3056912

[51] Abela AR, Dougherty SD, Fagen ED, Hill CJR, Chudasama Y. Inhibitory control deficits in rats with ventral hippocampal lesions. Cereb Cortex. 2013;23:1396–409. 22615141 10.1093/cercor/bhs121

[52] MacKillop J, et al. The latent structure of impulsivity: impulsive choice, impulsive action, and impulsive personality traits. Psychopharmacology. 2016;233:3361–70. 27449350 10.1007/s00213-016-4372-0 PMC5204128

[53] Diergaarde L, et al. Impulsive choice and impulsive action predict vulnerability to distinct stages of nicotine seeking in rats. Biol Psychiatry. 2008;63:301–8. 17884016 10.1016/j.biopsych.2007.07.011

[54] Paterson NE, Wetzler C, Hackett A, Hanania T. Impulsive action and impulsive choice are mediated by distinct neuropharmacological substrates in rat. Int J Neuropsychopharmacol. 2012;15:1473–87. 22094071 10.1017/S1461145711001635

[55] Abela AR, Chudasama Y. Noradrenergic α2A-receptor stimulation in the ventral hippocampus reduces impulsive decision-making. Psychopharmacology. 2014;231:521–31. 24062084 10.1007/s00213-013-3262-y

[56] Abela AR, Duan Y, Chudasama Y. Hippocampal interplay with the nucleus accumbens is critical for decisions about time. Eur J Neurosci. 2015;42:2224–33. 26121594 10.1111/ejn.13009 PMC5233438

[57] Mariano TY, et al. Impulsive choice in hippocampal but not orbitofrontal cortex-lesioned rats on a nonspatial decision-making maze task. Eur J Neurosci. 2009;30:472–84. 19656177 10.1111/j.1460-9568.2009.06837.x PMC2777256

[58] McHugh SB, Campbell TG, Taylor AM, Rawlins JNP, Bannerman DM. A role for dorsal and ventral hippocampus in inter-temporal choice cost-benefit decision making. Behav Neurosci. 2008;122:1–8. 18298243 10.1037/0735-7044.122.1.1 PMC2671844

[59] Sesia T, et al. Deep brain stimulation of the nucleus accumbens shell increases impulsive behavior and tissue levels of dopamine and serotonin. Exp Neurol. 2010;225:302–9. 20615406 10.1016/j.expneurol.2010.06.022

[60] Weafer J. Sex differences in neural correlates of inhibitory control. In Recent advances in research on impulsivity and impulsive behaviors (eds de Wit H, Jentsch JD). Springer International Publishing; Cham: 2020. 10.1007/7854_2020_146.

[61] Bayless DW, Darling JS, Daniel JM. Mechanisms by which neonatal testosterone exposure mediates sex differences in impulsivity in prepubertal rats. Hormones Behav. 2013;64:764–9. 10.1016/j.yhbeh.2013.10.003 24126137

[62] Darling JS, et al. Sex differences in impulsivity in adult rats are mediated by organizational actions of neonatal gonadal hormones and not by hormones acting at puberty or in adulthood. Behav Brain Res. 2020;395:112843. 32755634 10.1016/j.bbr.2020.112843 PMC7721484

[63] Jentsch JD, Taylor JR. Sex-related differences in spatial divided attention and motor impulsivity in rats. Behav Neurosci. 2003;117:76–83. 12619910 10.1037//0735-7044.117.1.76

[64] Panfil K, Deavours A, Kirkpatrick K. Effects of the estrous cycle on impulsive choice and interval timing in female rats. Hormones Behav. 2023;149:105315. 10.1016/j.yhbeh.2023.105315 PMC9974800 36669427

[65] Swalve N, et al. Sex-specific attenuation of impulsive action by progesterone in a go/no-go task for cocaine in rats. Psychopharmacology. 2018;235:135–43. 29018893 10.1007/s00213-017-4750-2 PMC5892199

[66] Carroll ME, Kohl EA, Johnson KM, LaNasa RM. Increased impulsive choice for saccharin during PCP withdrawal in female monkeys: influence of menstrual cycle phase. Psychopharmacology. 2013;227:413–24. 23344553 10.1007/s00213-012-2963-y PMC3656971

[67] Smith CT, Sierra Y, Oppler SH, Boettiger CA. Ovarian cycle effects on immediate reward selection bias in humans: a role for estradiol. J Neurosci. 2014;34:5468–76. 24741037 10.1523/JNEUROSCI.0014-14.2014 PMC3988406

[68] Hsu TM, et al. Hippocampus ghrelin receptor signaling promotes socially-mediated learned food preference. Neuropharmacology. 2018;131:487–96. 29191751 10.1016/j.neuropharm.2017.11.039 PMC5820148

[69] Suarez AN, Liu CM, Cortella AM, Noble EE, Kanoski SE. Ghrelin and orexin interact to increase meal size through a descending hippocampus to hindbrain signaling pathway. Biol Psychiatry. 2020;87:1001–11. 31836175 10.1016/j.biopsych.2019.10.012 PMC7188579

[70] Wee RWS, et al. Internal-state-dependent control of feeding behavior via hippocampal ghrelin signaling. Neuron. 2024;112:288–305.e7. 37977151 10.1016/j.neuron.2023.10.016 PMC12614488

[71] Barnstedt O, Mocellin P, Remy S. A hippocampus-accumbens code guides goal-directed appetitive behavior. Nat Commun. 2024;15:3196. 38609363 10.1038/s41467-024-47361-x PMC11015045

[72] Ibrahim KM, et al. Dorsal hippocampus to nucleus accumbens projections drive reinforcement via activation of accumbal dynorphin neurons. Nat Commun. 2024;15:750. 38286800 10.1038/s41467-024-44836-9 PMC10825206

[73] Engin E, Treit D. The role of hippocampus in anxiety: intracerebral infusion studies. Behav Pharmacol. 2007;18:365. 17762507 10.1097/FBP.0b013e3282de7929

[74] Ghasemi M, Navidhamidi M, Rezaei F, Azizikia A, Mehranfard N. Anxiety and hippocampal neuronal activity: Relationship and potential mechanisms. Cogn Affect Behav Neurosci. 2022;22:431–49. 34873665 10.3758/s13415-021-00973-y

[75] Pignatelli M, et al. Cooperative synaptic and intrinsic plasticity in a disynaptic limbic circuit drive stress-induced anhedonia and passive coping in mice. Mol Psychiatry. 2021;26:1860–79. 32161361 10.1038/s41380-020-0686-8 PMC7735389

[76] Chang C-H, Gean P-W. The Ventral Hippocampus Controls Stress-Provoked Impulsive Aggression through the Ventromedial Hypothalamus in Post-Weaning Social Isolation Mice. Cell Rep. 2019;28:1195–.e3. 31365864 10.1016/j.celrep.2019.07.005

[77] Chudasama Y, Doobay VM, Liu Y. Hippocampal-Prefrontal Cortical Circuit Mediates Inhibitory Response Control in the Rat. J Neurosci. 2012;32:10915–24. 22875926 10.1523/JNEUROSCI.1463-12.2012 PMC6621026

[78] Cabral DAR, et al. Food for thought: The relationship between poor eating habits, delay discounting, and quality of life in substance use recovery. Eat Behav. 2025;57:101972. 40174471 10.1016/j.eatbeh.2025.101972

[79] Hoptman MJ. Impulsivity and aggression in schizophrenia: a neural circuitry perspective with implications for treatment. CNS Spectr. 2015;20:280–6. 25900066 10.1017/S1092852915000206 PMC4441843

[80] Enticott PG, Ogloff JRP, Bradshaw JL. Response inhibition and impulsivity in schizophrenia. Psychiatry Res. 2008;157:251–4. 17916385 10.1016/j.psychres.2007.04.007

[81] Kaladjian A, Jeanningros R, Azorin J-M, Anton J-L, Mazzola-Pomietto P. Impulsivity and neural correlates of response inhibition in schizophrenia. Psychological Med. 2011;41:291–9. 10.1017/S0033291710000796 20406530

[82] Heerey EA, Robinson BM, McMahon RP, Gold JM. Delay discounting in schizophrenia. Cogn Neuropsychiatry. 2007;12:213–21. 17453902 10.1080/13546800601005900 PMC3746343

[83] Muir J, et al. Ventral hippocampal afferents to nucleus accumbens encode both latent vulnerability and stress-induced susceptibility. Biol Psychiatry. 2020;88:843–54. 32682566 10.1016/j.biopsych.2020.05.021

[84] Mole TB, Mak E, Chien Y, Voon V. Dissociated accumbens and hippocampal structural abnormalities across obesity and alcohol dependence. 10.1093/ijnp/pyw039. 10.1093/ijnp/pyw039 PMC5043646 27207916

[85] Bossert JM, et al. Role of projections from ventral subiculum to nucleus accumbens shell in context-induced reinstatement of heroin seeking in rats. Psychopharmacology. 2016;233:1991–2004. 26344108 10.1007/s00213-015-4060-5 PMC4781679

[86] Rogers JL, See RE. Selective inactivation of the ventral hippocampus attenuates cue-induced and cocaine-primed reinstatement of drug-seeking in rats. Neurobiol Learn Mem. 2007;87:688–92. 17337218 10.1016/j.nlm.2007.01.003 PMC1896086

[87] Mikell CB, et al. The hippocampus and nucleus accumbens as potential therapeutic targets for neurosurgical intervention in schizophrenia. Stereotact Funct Neurosurg. 2009;87:256–65. 19556835 10.1159/000225979 PMC2836942

[88] Tseng KY, Chambers RA, Lipska BK. The neonatal ventral hippocampal lesion as a heuristic neurodevelopmental model of schizophrenia. Behav Brain Res. 2009;204:295–305. 19100784 10.1016/j.bbr.2008.11.039 PMC2735579

[89] McCollum LA, Walker CK, Roche JK, Roberts RC. Elevated excitatory input to the nucleus accumbens in schizophrenia: a postmortem ultrastructural study. Schizophr Bull. 2015;41:1123–32. 25817135 10.1093/schbul/sbv030 PMC4535638

[90] Chang C-Y, et al. The role of dorsomedial striatum and parvalbumin interneurons in schizophrenia-related cognitive deficits: insights from disrupted strategic decision-making in Akt1 heterozygous mice. Transl Psychiatry. 2025;15:362. 41053102 10.1038/s41398-025-03541-x PMC12500987

[91] Xu M, Wong AHC. GABAergic inhibitory neurons as therapeutic targets for cognitive impairment in schizophrenia. Acta Pharm Sin. 2018;39:733–53. 10.1038/aps.2017.172 PMC5943898 29565038

[92] Nakazawa K, et al. GABAergic interneuron origin of schizophrenia pathophysiology. Neuropharmacology. 2012;62:1574–83. 21277876 10.1016/j.neuropharm.2011.01.022 PMC3090452

[93] Lodge DJ, Behrens MM, Grace AA. A loss of parvalbumin-containing interneurons is associated with diminished oscillatory activity in an animal model of schizophrenia. J Neurosci. 2009;29:2344–54. 19244511 10.1523/JNEUROSCI.5419-08.2009 PMC2754752

[94] Barlow RL, et al. Ventral Striatal D2/3 receptor availability is associated with impulsive choice behavior as well as limbic corticostriatal connectivity. Int J Neuropsychopharmacol. 2018;21:705–15. 29554302 10.1093/ijnp/pyy030 PMC6030945

[95] Moreno M, et al. Divergent effects of D2/3 receptor activation in the nucleus accumbens core and shell on impulsivity and locomotor activity in high and low impulsive rats. Psychopharmacology. 2013;228:19–30. 23407782 10.1007/s00213-013-3010-3 PMC3676742

[96] Heal DJ, et al. Dopamine and μ-opioid receptor dysregulation in the brains of binge-eating female rats—possible relevance in the psychopathology and treatment of binge-eating disorder. J Psychopharmacol. 2017;31:770–83. 28376679 10.1177/0269881117699607

[97] Giacomini JL, Geiduschek E, Selleck RA, Sadeghian K, Baldo BA. Dissociable control of μ-opioid-driven hyperphagia vs. food impulsivity across subregions of medial prefrontal, orbitofrontal, and insular cortex. Neuropsychopharmacol. 2021;46:1981–9. 10.1038/s41386-021-01068-5 PMC8429588 34226656

[98] Olmstead MC, Ouagazzal A-M, Kieffer BL. Mu and delta opioid receptors oppositely regulate motor impulsivity in the signaled nose poke task. PLoS ONE. 2009;4:e4410. 19198656 10.1371/journal.pone.0004410 PMC2635474

[99] Wiskerke J, et al. Opioid receptors in the nucleus accumbens shell region mediate the effects of amphetamine on inhibitory control but not impulsive choice. J Neurosci. 2011;31:262–72. 21209211 10.1523/JNEUROSCI.4794-10.2011 PMC6622756

