# Supplementary material for: A ventral hippocampus to nucleus accumbens pathway regulates impulsivity in male but not female rats
Source: Neuropsychopharmacology. 2026 Jun 26;51(10):1779–89. doi: 10.1038/s41386-026-02477-0 (PMC13487274; doi:10.1038/s41386-026-02477-0)
Supplement: Supplementary file 1 [file 41386_2026_2477_MOESM1_ESM.pdf]

Supplementary Figure 1.

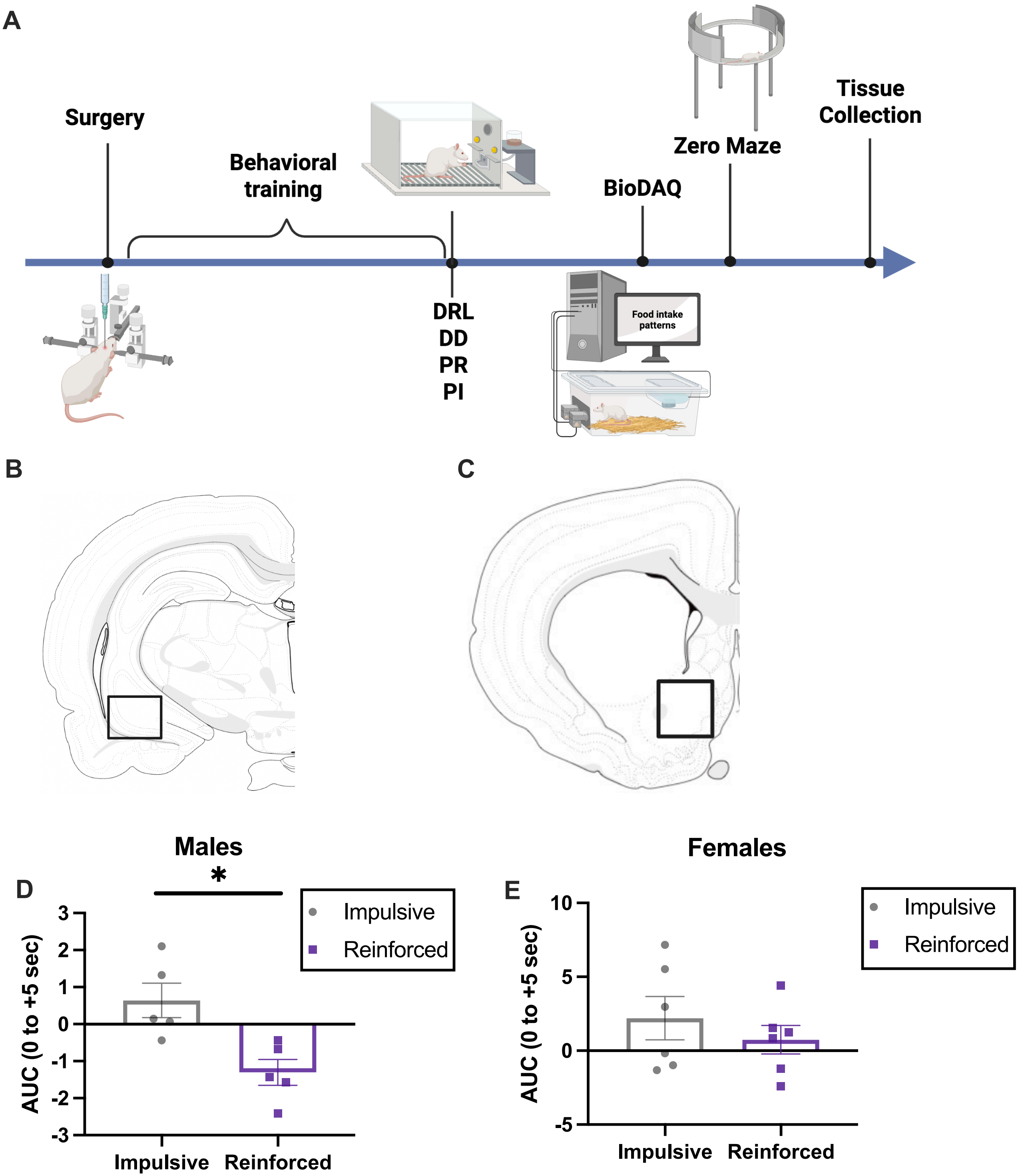

Supplementary Figure 1. Surgical procedures, histological criteria, and Changes in calcium-dependent activity after lever pressing. (A) Timeline of surgical procedure and behavioral experiments. **(B)** Inclusion criteria for CA1v injections. **(C)** Inclusion criteria for ACBsh injections. **(D)** Ca^{2+}-dependent activity in the CA1v is significantly lowered in the 5s after a non-impulsive lever press, relative to an impulsive press in males ($n = 5$, paired two-tail t-test, $df = 4$, $p = .0432$) but not **(E)** in females ($n = 6$, paired two-tail t-test, $df = 5$, $p = .3289$). (Data are means ± SEM; $*p<0.05$).

Supplementary Figure 2.

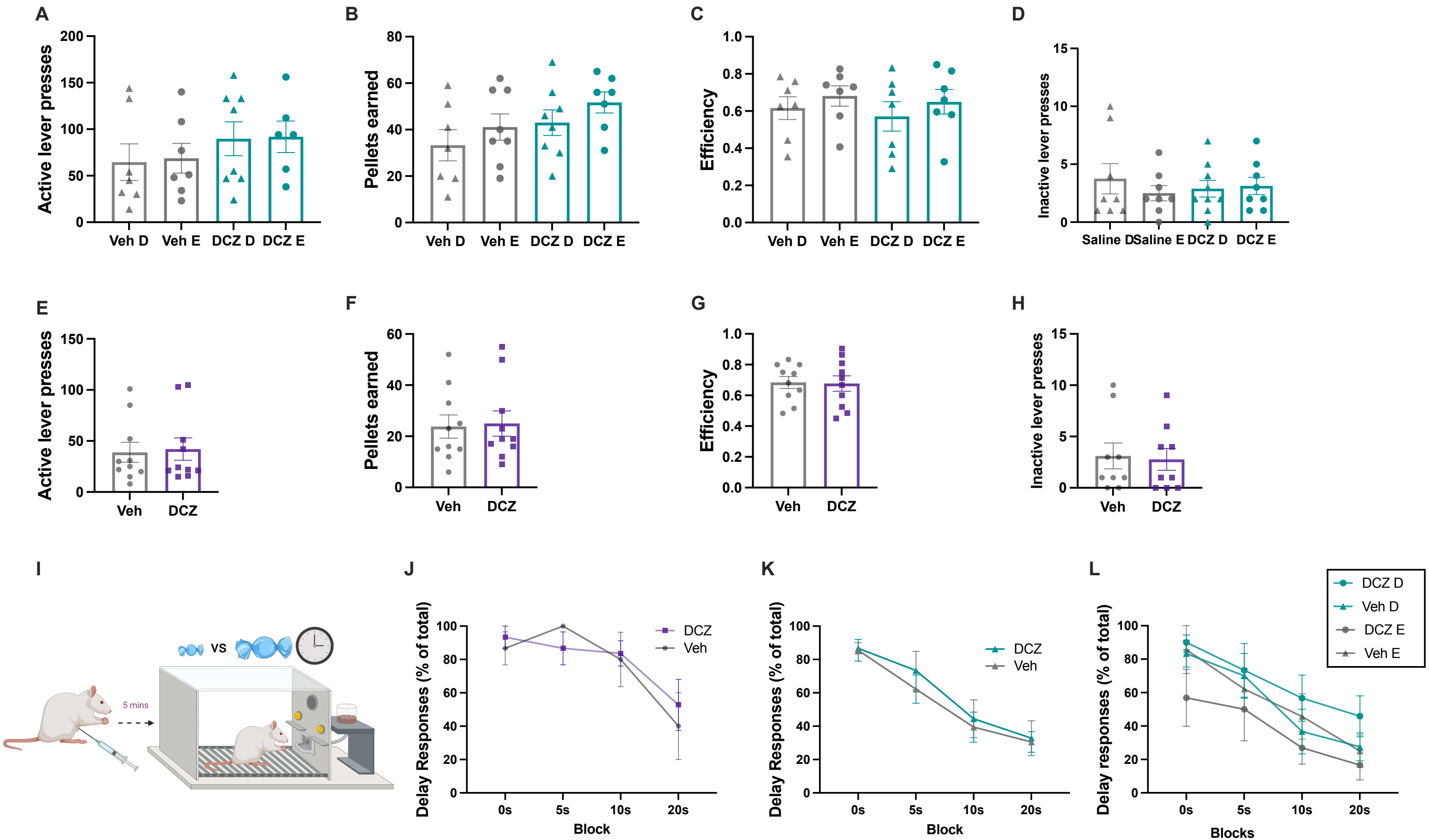

Supplementary Figure 2. Silencing ACBsh-projecting CA1v neurons does not affect impulsive choice in delay discounting, nor does estrus stage influence impulsive action or choice. (A) Active lever presses (one-way ANOVA, DCZ E n = 6, Veh E n = 7, DCZ D = 8, Veh D n = 7, f = .6058, df = 3,24 , p =.6177), **(B)** rewards earned (one-way ANOVA, DCZ E n = 7, Veh E n = 8, DCZ D = 8, Veh D n = 7, f = 1.665, df = 3,26 , p =.1989), **(C)** efficiency (one-way ANOVA, DCZ E n = 7, Veh E n = 7, DCZ D = 7, Veh D n = 7, f = .5081, df = 3,24 , p =.6804) and **(D)** inactive lever presses (one-way ANOVA, DCZ E n = 8, Veh E n = 8, DCZ D = 9, Veh D n = 8, f = .5663, df = 3,29 , p =.7955) are unaffected by chemogenetic inhibition, even when accounting for estrus cycle. **(E)** Active lever presses (n = 10 , paired two-tail t-test, df = 9, p =.6187), **(F)** rewards earned (n = 10, paired two-tail t-test, df = 9, p =.7045), **(G)** efficiency (n = 10 , paired two-tail t-test, df =9 , p =.8985) and **(H)** inactive lever presses (n = 10 , paired two-tail t-test, df =9 , p =.7245) are unaffected by DCZ administration in control DREADDs animals. **(I)** Schematic representing the delay discounting procedure. **(J)** Delay discounting is not affected by chemogenetic inhibition in males (Mixed-effects analysis, n = 6, block x treatment p =.6751, block p = .0125, treatment p = .7965, no differences on multiple comparisons). **(K)** Delay discounting is not affected by chemogenetic inhibition in females (2-way ANOVA, n = 9, block x treatment p =.8324, block p <0.0001, treatment p = 6176, no differences on multiple comparisons). **(L)** Delay discounting is not affected by chemogenetic inhibition in females, even when accounting for estrus cycle (Mixed-effects analysis, DCZ E n = 5, Veh E n = 6, DCZ D = 6, Veh D n = 6, block x treatment p =.8104, block p = .0001, treatment p = .3008, no differences on multiple comparisons). (Data are means ± SEM; *p<0.05).

Supplementary Figure 3.

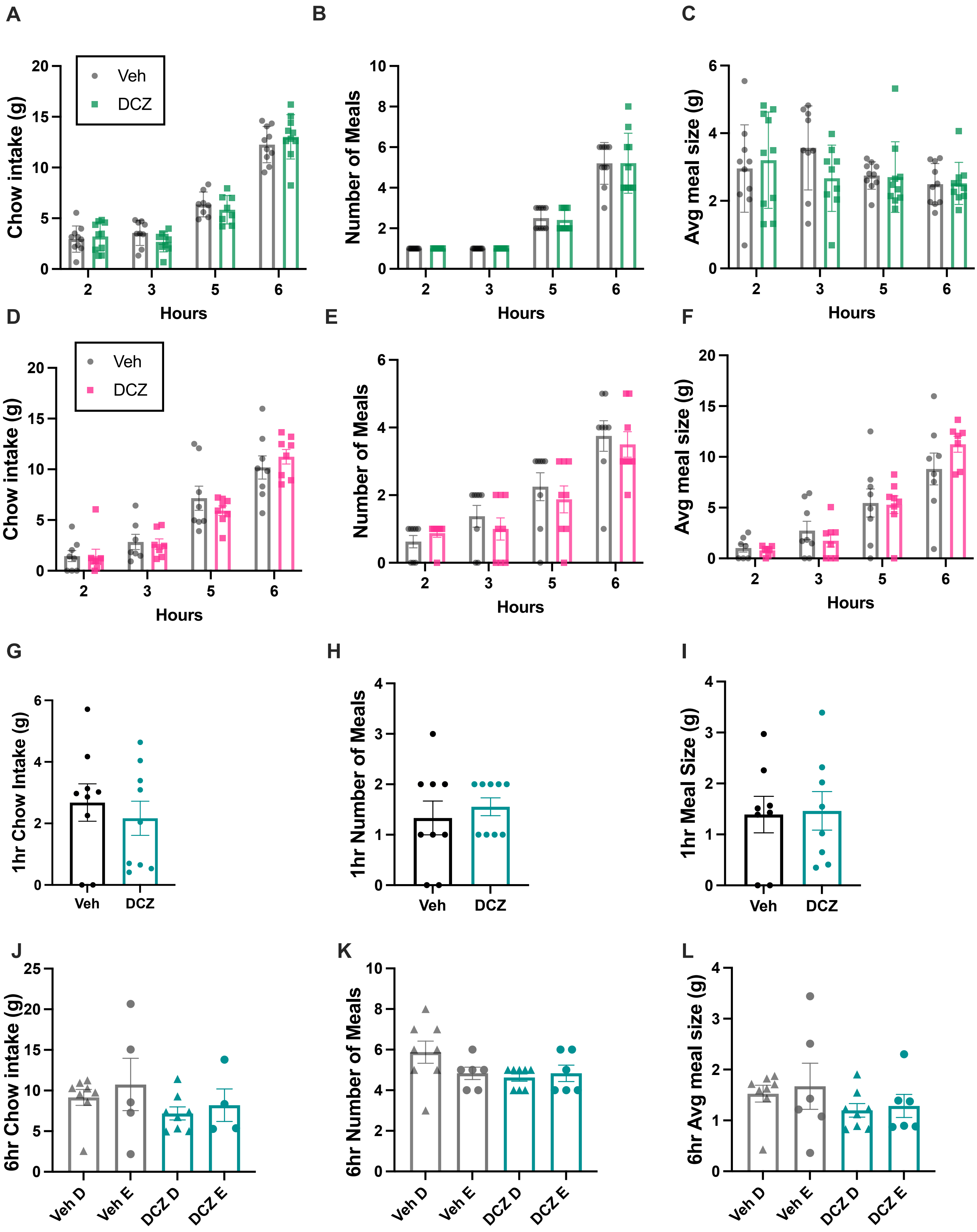

Supplementary Figure 3. Chemogenetic inhibition of ACBsh-projecting CA1v neurons pathway does not affect home cage intake in males and females. (A) Total chow intake (Mixed-effects analysis, n = 8, time x treatment p =.3199, time p < .0001, treatment p = .7782, no differences on multiple comparisons) **(B)** number of meals (2-way ANOVA, n =10 , time x treatment p =.9198, time p < .0001, treatment p = .8735 , no differences on multiple comparisons) and **(C)** average meal size (Mixed-effects analysis, n =10 , time x treatment p =.3250, time p = .2065, treatment p = .4665, no differences on multiple comparisons) were unaffected by chemogenetic silencing at all timepoints for standard chow. Likewise, **(D)** Total chow intake (Mixed-effects analysis, n = 8, time x treatment p = .3131, time p < .0001, treatment p = .8683, no differences on multiple comparisons), **(E)** number of meals (Mixed-effects analysis, n =8 , time x treatment p =.4219, time p < .0001, treatment p = .6466, no differences on multiple comparisons), and **(F)** average meal size (Mixed-effects analysis, n =8 , time x treatment p =.1358, time p < .0001, treatment p = .9091, no differences on multiple comparisons) were also unaffected by chemogenetic silencing at all timepoints for HFHS chow. No differences were seen in **(G)** total chow intake (n = 9, paired two-tail t-test, df = 8, p =.5777) **(H)** number of meals (n = 9, paired two-tail t-test, df = 8, p =.5943) and **(I)** average meal size (n = 8, paired two-tail t-test, df = 7, p =.8967) with chemogenetic silencing at the 1hr timepoint for standard chow in females. Similarly, there were no differences in **(J)** total chow intake (One-way ANOVA, DCZ E n = 4, Veh E n = 5, DCZ D = 8, Veh D n = 8, f= .8526 df = 3,21, p =.4809) **(K)** number of meals (One-way ANOVA, DCZ E n = 5, Veh E n = 6, DCZ D = 8, Veh D n = 8, f= 2.253 df = 3,24, p =.1080) and **(L)** average meal size (One-way ANOVA, DCZ E n = 6, Veh E n = 6, DCZ D = 8, Veh D n = 8, f= .7640 df = 3,24, p =.5254) with chemogenetic silencing at the 6r timepoint for standard chow in females when accounting for estrus stage. (Data are means ± SEM; *p<0.05, ***p<0.001).

Supplemental Methods

Estrus tracking

To identify estrous stage, vaginal cytology was investigated by vaginal lavage[1–4]. Cytology samples were collected in 0.9% sterile saline (Teknova) 1.5 hr prior to the start of the dark cycle. Vaginal smears were examined at 10x using a Nikon 80i (Nikon DS-QI1,1280X1024 resolution, 1.45 megapixel). Stages were assigned at the same time each day to minimize the incidence of transitional and 'missed' stages and were based on vaginal cell morphology (presence or absence of epithelial cells, cornified cells, and leucocytes) as described in [1].

Surgical procedures

For all surgical procedures, rats were anesthetized and sedated via either intramuscular injections of ketamine (90 mg/kg), xylazine (2.8 mg/kg), and acepromazine (0.72 mg/kg), or 4% isoflurane in oxygen. Animals were shaved, surgical site was prepped with iodine and ethanol swabs, and animals were placed in a stereotaxic apparatus for stereotaxic injections. For fiber photometry surgeries, animals were given subcutaneous injections of buprenorphine SR (0.65 mg/kg) during surgery as an analgesic. For all other surgeries, rats were also given analgesic (subcutaneous injection of ketoprofen [5mg/kg] or meloxicam [1mg/kg]) after surgery and once daily for 3 subsequent days thereafter. All rats recovered for at least one-week post-surgery prior to experiments.

Viral injections

Viruses were delivered using a microinfusion pump (Chemyx, Stafford, TX, USA) connected to a 33-gauge microsyringe injector attached to a PE20 catheter and Hamilton syringe. Flow rate was calibrated and set to 5 µl/min. Injectors were left in place for 2 min postinjection. Following injections, animals were either sutured or surgically implanted with a cannula. All experimental procedures occurred 21 days post surgery to allow for viral transduction and expression. Successful transduction was confirmed postmortem in all animals via IHC staining using immunofluorescence-based antibody amplification to enhance the fluorescence followed by manual quantification under epifluorescence illumination using a Nikon 80i (Nikon DS-QI1,1280X1024 resolution, 1.45 megapixel). All injection volumes were based on previous work[5–7].

For recording of $CA^{2}+$ activity, 400nl of AAV9-hSyn-GCaMP7s-WPRE (Cat. no. 104487-AAV9, Addgene) was unilaterally injected at the following coordinates: CA1v: –4.9 mm

anterior/posterior (AP), +-4.8 mm medial/lateral (ML), −7.8 mm dorsal/ventral (DV) (0 reference point at bregma for ML, AP, 0 reference point at skull site for DV) ACBsh: +1.2 mm anterior/posterior (AP), +-1.0 mm medial/lateral (ML), −6.75 mm dorsal/ventral (DV) (0 reference point at bregma for ML, AP, 0 reference point at dura for DV). An optic fiber was then implanted at the injection site as described below. Following viral infusions, a fiber-optic cannula (Doric Lenses Inc, Quebec, Canada; flat 400-μm core, 0.48 numerical aperture [NA]) was implanted in the CA1v at the following coordinates: CA1v: −4.9 mm anterior/posterior (AP), +-4.8 mm medial/lateral (ML), −7.8 mm dorsal/ventral (DV) (0 reference point at bregma for ML, AP, 0 reference point at skull site for DV). The optic fibers were then affixed to the skull with jeweler's screws, instant adhesive glue, and dental cement. All subjects were given 1 week to recover from surgery prior to experiments.

For chemogenetic inhibition of CA1v neurons projecting to the ACBsh via DREADDS, an AAV2-hSyn-DIO-hM4D(Gi)-mCherry, (DIO-DREADDs; Cat. no. 44362-AAV2, Addgene) was bilaterally injected at the following coordinates: −4.9 mm anterior/posterior (AP), +-4.8 mm medial/lateral (ML), −7.8 mm dorsal/ventral (DV) (0 reference point at bregma for ML, AP, 0 reference point at skull site for DV). Additionally, an AAV2(retro)-hSYN1-eGFP-2A-iCre-WPRE; (Retro-cre; Cat. no. VB4855, Vector Biolabs) was bilaterally injected at the following coordinates, to allow for targeted inhibition of ACBsh-projecting CA1v neurons: +1.2 mm anterior/posterior (AP), +-1.0 mm medial/lateral (ML), −6.75 mm dorsal/ventral (DV) (0 reference point at bregma for ML, AP, 0 reference point at dura for DV). The injection volume was 300 nl/site.

For pathway-specific chemogenetic inhibition of ACBsh neurons receiving glutamatergic projections from the CA1v, AAV8-ATLAS$_{cre}$ and pAAV-DIO0BACE-HA (ATLAScre; Gift from the Arnold Lab [7]; available on Addgene, ID 232351 and ID232353) was bilaterally injected at a 1:1 ratio in the following coordinates: −4.9 mm anterior/posterior (AP), +-4.8 mm medial/lateral (ML), −7.8 mm dorsal/ventral (DV) (0 reference point at bregma for ML, AP, 0 reference point at skull site for DV). Additionally, AAV2-hSyn-DIO-hM4D(Gi)-mCherry, (DIO-DREADDs; Cat. no. 44362-AAV2, Addgene) was bilaterally injected at the following coordinates: +1.2 mm anterior/posterior (AP), +-1.0 mm medial/lateral (ML), −6.75 mm dorsal/ventral (DV) (0 reference point at bregma for ML, AP, 0 reference point at dura for DV). In the presence of Cre-driven transsynaptically from CA1v neurons, this later injection drives expression of the DREADDs transgene, allowing us to target ACBsh neurons that receive projections from the

CA1v specifically for chemogenetic inhibition. The injection volume for both viruses was 300-400 nl/site.

Drug preparation

For chemogenetic inhibition, 1 ml/kg deschloroclozapine (DCZ) (100 µg/kg) or vehicle (1% DMSO in 99% saline) is administered intraperitoneally through a syringe, 5 mins prior to behavioral testing. Doses and concentration of DCZ and vehicle were based on previous work [8].

Immunohistochemistry

Rats were anesthetized and sedated with a ketamine (90 mg/kg)/xylazine (2.8 mg/kg)/acepromazine (0.72 mg/kg) cocktail, then transcardially perfused with 0.9% sterile saline (pH 7.4) followed by 4% paraformaldehyde (PFA) in 0.1 M borate buffer (pH 9.5; PFA). Brains were dissected out and post-fixed in PFA with 15% sucrose for 24 h, then flash frozen in isopentane cooled in dry ice. Brains were sectioned to 30-µm thickness on a freezing microtome. Sections were collected in 5 series and stored in antifreeze solution at –20 °C until further processing.

General fluorescence IHC labeling procedures were performed for histological confirmation. Primary antibodies and dilutions used for each experiment are detailed below. Antibodies were prepared in 0.02 M potassium phosphate-buffered saline (KPBS) solution containing 0.2% bovine serum albumin and 0.3% Triton X-100 at 4 °C overnight. After thorough washing with 0.02 M KPBS, sections were incubated in secondary antibody solution. All secondary antibodies were obtained from Jackson Immunoresearch and used at 1:500 dilution at 4 °C, with overnight incubations (Jackson Immunoresearch; West Grove, PA, USA). Sections were mounted and coverslipped using 50% glycerol in 0.02 M KPBS and the edges were sealed with clear nail polish. Photomicrographs were acquired using either a Nikon 80i (Nikon DS-QI1,1280X1024 resolution, 1.45 megapixel) under epifluorescence or darkfield illumination.

Histology

Sections were prepared and imaged as described above. The following primary antibodies were used for histological confirmation of viral injection sites: rabbit anti-RFP (1:2000, Rockland Inc., Limerick, PA, USA), chicken anti-GFP (1:1000, Cat. no. ab13970, Abcam, Cambridge, UK), and ALFA-TAG (1:1000, Cat No: N1502, NanoTag Biotechnologies, Gottingen, Germany). For all viral experiments, histological confirmation for inclusion in subsequent statistical analyses

was based on identification of injections sites confined with the CA1v and ACBsh, as observed using darkfield microscopy. Included photomicrograph images captures all fluorescent labeling present on those coronal sections. Please refer to Supplementary Figure 1B, C to view boundaries necessary for inclusion in studies for injection sites. On average, n=3 animals were excluded for failing histological confirmation per cohort (average cohort n = 11).

Cell counting and quantification

Sections were prepared as described above. The following primary antibodies were used for identification of inhibitory interneurons within the ACBsh: rabbit anti-parvalbumin (1:500, Cat. No. ab 11427,Abcam, Cambridge, UK), mouse anti-somatostatin (1:500, ThermoFisher Scientific, Waltham, MA, USA), and guinea pig anti-RFP (1:5000, Cat. No. 390 004, SYSY Antibodies, Gottingen, Germany). Colocalization of RFP+ cells – i.e. those tagged for pathway specific chemogenetic inhibition – and individual inhibitory interneuron markers were quantitatively analyzed in a subset of rats (n= 3-4 sections/rat; rat n=3). The reported value was calculated by first determining the number of labeled cells in each section and then averaging these values across sections within each animal, followed by averaging across animals. The percentage of colocalization of RFP+ cells with inhibitory interneuron markers was then found using the following formula: ((# cells + for RFP and somatostatin OR paravalbumin)/(Total # of RFP+ cells)) * 100.

Supplementary References

1. Liu, C. M. *et al.* Sex Differences and Estrous Influences on Oxytocin Control of Food Intake. *Neuroscience* **447**, 63–73 (2020).
2. Becker, J. B. *et al.* Strategies and Methods for Research on Sex Differences in Brain and Behavior. *Endocrinology* **146**, 1650–1673 (2005).
3. Ekambaram, G. Comparative Study on the Estimation of Estrous Cycle in Mice by Visual and Vaginal Lavage Method. *JCDR* https://doi.org/10.7860/JCDR/2017/23977.9148 (2017) doi:10.7860/JCDR/2017/23977.9148.

4. McLean, A. C., Valenzuela, N., Fai, S. & Bennett, S. A. L. Performing Vaginal Lavage, Crystal Violet Staining, and Vaginal Cytological Evaluation for Mouse Estrous Cycle Staging Identification. *Journal of Visualized Experiments (JoVE)* e4389 (2012) doi:10.3791/4389.
5. Noble, E. E. *et al*. Hypothalamus-hippocampus circuitry regulates impulsivity via melanin-concentrating hormone. *Nat Commun* **10**, 4923 (2019).
6. Décarie-Spain, L. *et al*. Ventral hippocampus-lateral septum circuitry promotes foraging-related memory. *Cell Reports* **40**, (2022).
7. Rivera, J. F. *et al*. ATLAS: a rationally designed anterograde transsynaptic tracer. *Nat Methods* **22**, 1101–1111 (2025).
8. Subramanian, K. S. *et al*. Hypothalamic melanin-concentrating hormone neurons integrate food-motivated appetitive and consummatory processes in rats. *Nat Commun* **14**, 1755 (2023).
